# Validation of the Thai version of the Prolapse and Incontinence Knowledge Questionnaire (PIKQ)

**DOI:** 10.1007/s00192-021-05020-5

**Published:** 2021-12-22

**Authors:** Wanchat Komon, Jittima Manonai, Athasit Kijmanawat, Chatchawan Silpakit, Bhatarachit Tunkoon, Ketkaew Jengprasert, Sirirat Sarit-apirak

**Affiliations:** 1 Department of Obstetrics & Gynaecology, Faculty of Medicine Ramathibodi Hospital, Mahidol University, 270 Rama VI Road, Ratchathewi, Bangkok, 10400 Thailand; 2 Department of Psychiatry, Faculty of Medicine Ramathibodi Hospital, Mahidol University, Bangkok, Thailand; 3 Obstetrics and Gynecology Nursing Division, Faculty of Medicine Ramathibodi Hospital, Mahidol University, Bangkok, Thailand; 4 Somdech Phra Debaratana Medical Center, Faculty of Medicine Ramathibodi Hospital, Mahidol University, Bangkok, Thailand

**Keywords:** Knowledge, Questionnaire, Pelvic organ prolapse, Urinary incontinence, Validity, Reliability

## Abstract

**Introduction and hypothesis:**

The Prolapse and Incontinence Knowledge Questionnaire (PIKQ) was developed and validated to assess women’s knowledge regarding etiology, diagnosis and treatment of pelvic organ prolapse (POP) and urinary incontinence (UI). We aimed to translate and validate a Thai version of the PIKQ to use as a tool to evaluate knowledge of POP and UI among Thai-speaking women.

**Methods:**

The English PIKQ, which comprises the PIKQ-POP and PIKQ-UI sections, was translated into Thai. Psychometric properties of the final version of the Thai PIKQ were tested for content validity, construct validity, internal consistency and test-retest reliability among 168 women attending a gynecology clinic and 150 nurses.

**Results:**

Regarding content validity of the final Thai PIKQ, the number of missing items was 0. Participants in the nurse group were more likely than those in the patient group to select the correct answer for all items for the POP scale and UI scale (*P* < 0.001). For internal consistency testing, Cronbach’s alpha coefficient was 0.745 for the PIKQ-POP and 0.754 for the PIKQ-UI scales, suggesting that the items had relatively high internal consistency. The item-total correlation values ranged from 0.204 to 0.539, showing an adequate correlation of each item with the scale overall. The correlation coefficients between the test and retest for PIKQ-POP and PIKQ-UI were 0.685 and 0.735, respectively (*P* < 0.001).

**Conclusions:**

The Thai PIKQ is a simple instrument which shows good validity and high reliability and could be a useful tool for assessing knowledge regarding POP and UI in clinical practice.

## Introduction

Pelvic floor dysfunction (PFD), including a wide variety of clinical conditions, is common and affects women of all ages worldwide [1–3]. Among community-dwelling women in low and middle-income countries, pelvic organ prolapse (POP) and female urinary incontinence (UI) are the leading PFD problems with the pooled prevalence of 15% (95% CI 10–20%) and 30% (95% CI 25–35%), respectively [3]. The global prevalence of POP varies from 2 to 50% [3], while prevalence estimates for UI vary from 10 to 58% in women living in community settings and from 50 to 84% among long-term care residents [4]. PFD has a negative impact on the quality of life of many women. Previous reports demonstrated that women suffer significant physical and emotional distress from POP and UI, including depression, loss of self-esteem, social isolation and poor sleep quality [5, 6].

Effective treatment options for PFD may consist of behavioral modification and conservative and surgical treatment, including a preventive approach in asymptomatic women. However, many women do not have information or knowledge regarding their condition and treatment options [7]. The impact of women’s attitude and their understanding of the nature of the pelvic floor condition could affect health-seeking behavior and cause delay in receiving appropriate care. A previous study reported that < 50% of incontinent women seek medical care [8]. The delay in care seeking may cause depression, anxiety, impaired quality of life and sexual dysfunction as well as restrict their ability to engage in leisure activities and social participation [9]. A recent systematic review concluded that most women have a gap in their knowledge of pelvic floor dysfunction, including risk factors and treatment options [10].

Although there are reports of patient’s perception or patient-reported outcomes about pelvic floor dysfunction, such as symptoms or their impacts on quality of life in the literature, validated instruments assessing patient knowledge regarding PFD are lacking. Thus, a 14-item Incontinence Quiz was designed to assess patient knowledge concerning UI [11]. Still, the psychometric properties of this questionnaire have not been established. In addition, this questionnaire does not assess patient knowledge related to POP. Later on, the Prolapse and Incontinence Knowledge Questionnaire (PIKQ) was established to assess knowledge regarding etiology, diagnosis and treatment of both POP and UI [12]. This questionnaire comprises 2 sections with 12 items in each section and has been demonstrated as a validated and reliable evaluation tool to identify the knowledge gap [12]. The PIKQ has been widely used in clinical and research settings. Since there is no validated Thai version of this particular questionnaire, the authors aimed to translate and validate a Thai version of the PIKQ to use it as a tool to evaluate knowledge of POP and UI among Thai-speaking women.

## Materials and methods

This study was conducted at a university hospital in Bangkok, Thailand, from December 2020 to June 2021. The study protocol was approved by the Human Research Ethics Committee, Faculty of Medicine, Ramathibodi Hospital, Mahidol University (protocol no. MURA2020/1960).

The PIKQ is a self-completed questionnaire for assessing patient knowledge regarding both POP and UI, which demonstrates good psychometric property in terms of validity and reliability [7]. It contains two distinct 12-item scales—PIKQ-POP and PIKQ-UI—to assess knowledge regarding epidemiology, pathogenesis, diagnosis and treatment of POP and UI, respectively. The answer options available are “agree,” “disagree” or “don’t know.” The score range for each item was 0 (incorrect or unknown answer) to 1 (correct answer). Total PIKQ-POP and PIKQ-UI scale scores are computed by summing the number of correct responses within each scale. In each scale, the minimum score is 0 and the maximum is 12. Permission to use the questionnaire was obtained from the original authors before submitting the research protocol.

The study was divided into two stages. Stage 1 addressed the translation of the PIKQ into Thai and its cross-cultural adaptation. Stage 2 comprised the testing of the Thai PIKQ’s psychometric properties and its validation in different settings.

### Stage 1 translation

The translation processes based on the cross-cultural adaptation process for patient-reported outcome measures [13] were conducted in the following manner:

1. Initial translation (English to Thai) was independently undertaken by two Thai-speaking translators, who were experienced in translating health questionnaires. They were fluent in English and were aware of the study aims.
2. After the most appropriate wording had been selected in the common Thai version, two translators with bilingual proficiency who had no prior knowledge of the PIKQ individually translated the Thai version into English.
3. During the translation team meeting, the original version of the PIKQ and two back-translated versions were compared and reconciled, and the final draft of the Thai version was produced.
4. A pretest was performed in 20 women presenting to a gynecology clinic to ensure that the wording of all items was simple for them to understand. Making adjustments based on their feedback, while maintaining the meaning and content of original items, the final Thai version of PIKQ was produced and ready to be used for this study.

### Stage 2 psychometric property testing

The validity and reliability of the instrument were assessed in a cross-sectional analytical study conducted between January 2021 and April 2021. Eligible participants were women aged 20–80 years old who communicated fluently in Thai, were willing to participate and provided signed written informed consent. The final Thai-version PIKQ was distributed to (1) patients attending a general gynecology clinic, Ramathibodi Hospital (the patient group), and (2) registered nurses who had been working in Ramathibodi Hospital (the nurse group). Participants were excluded from the study if they withdrew or did not complete the study. After 10–14 days, the participants were asked to redo the same questionnaire. No money or any kind of compensation was provided.

The Thai PIKQ questionnaire was examined according to the quality criteria for measurement properties of health status questionnaires [14].

1. Face/content validity: An assessment of whether the questionnaire made sense when being measured and used in the clinical area was performed. The indicators were response rates and level of missing data.
2. Construct validity: To provide evidence of construct validity, the ability to differentiate between the different participant groups was tested. This property was examined by comparing PIKQ-POP and PIKQ-UI scores between the patient group and nurse group. Higher scores would also predicted from the nurse group compared to the patient group. In addition, construct validity was examined separately for PIKQ-POP and PIKQ-UI using confirmatory factor analysis (CFA).
3. Internal consistency (reliability): The correlation between the items was assessed by determining the Cronbach’s alpha coefficient and the total score without it (item-total correlations).
4. Stability: The test-retest reliability of this questionnaire was measured by having participants complete the same test twice, with a 2-week interval between the initial and second tests. All participants in the nurse group were invited to participate in the retest study.

### Statistical analyses

All statistics were performed using STATA 17.0/SE (StataCorp), and the results were considered statistically significant at *p* < 0.05. Regarding validity of the Thai-PIKQ, the content validity was evaluated using the missing value. Then, the construct validity was examined separately for PIKQ-POP and PIKQ-UI. Mann-Whitney U test was administered to compare the total scale scores of the PIKQ-POP and PIKQ-UI across participants in the patient and the nurse groups. Subsequently, confirmatory factor analysis (CFA) was performed with the maximum likelihood estimator to test whether the data fit a hypothesized measurement model. We included the following fit statistics in the analyses: standardized and unstandardized factor loadings (SFL and FL), chi-square (χ2), degree of freedom (df), comparative fit index (CFI), Tucker-Lewis index (TLI), root mean square error of approximation (RMSEA) and standardized root mean square residual (SRMR) [15]. The criteria for an acceptable model fit were CFI ≥ 0.95, TLI ≥ 0.95, RMSEA ≤ 0.06 [16] and SRMR ≤ 0.08 [17].

Regarding the Thai-PIKQ’s reliability, the internal consistency was determined using Cronbach’s alpha, with values ≥ 0.70 indicating good internal consistency [18]. Item-total correlations were correspondingly analyzed, considering cutoff values > 0.20 or 0.30 [19]. Test-retest reliability was measured using the intraclass correlation coefficient (ICC), with values between 0.5–0.9 considered moderate-to-good and > 0.9 excellent reliability [20].

Validation of a questionnaire with 24 items requires a sample size of 240 to satisfy the 10 subjects per item ratio [21, 22]. Sample size estimation adjusting for 20% drop-out rate resulted in 288 participants required. Based on the appropriate sample size for factor analysis, a sample size of at least 200 is adequate in most cases involving no more than 40 items [23, 24].

## Results

### Stage 1: translation and cross-cultural adaptation

The translation and cross-cultural adaptation process of the Thai version of PIKQ was completed and the final version was developed (online appendix). The reconciliation of the forward-translations and the harmonization across two back-translations were performed by two key in-country experts (JM and CS). After cognitive debriefing interviews of 20 women attending a general gynecology clinic, no changes were applied according to the respondents’ suggestions, and they stated that all items were clearly understandable.

### Stage 2: instrument validity and reliability assessment

#### Participants

The PIKQ was administered to 170 patients visiting a general gynecology clinic. Two patients who agreed to participate refused to return the questionnaire, while 168 completed the questionnaire (98.8% response rate). For the nurse group, the questionnaire was administered to 150 nurses who have been working in Faculty of Medicine of Ramathibodi Hospital; all responses were received (100.0% response rate). In summary, among 320 women who received the Thai PIKQ questionnaire, 318 returned the completed form, resulting in the response rate of 99.4%.

A total of 318 women (168 from the patient group and 150 from the nurse group) were included in the analyses. The median age of the participants was 42 years (minimum–maximum: 20–79 years) and 32 years (minimum–maximum: 22–57 years) in the patient group and the nurse group, respectively. While 150 (100%) nurses had received graduate degrees, the level of education in the patient group ranged from graduate study (64.7%) to a primary school education (4.3%).

#### Face and content validity

Cognitive debriefing interviews were performed for face validity analysis. All participants reported that all the items and the format were comprehensible, there were no ambiguities, and there was no need for any changes. Regarding content validity, the number of missing items was 0. Missing data resulting from respondents’ misunderstanding or misperception of any item were not reported.

#### Construct validity

The construct validity of each scale was verified by comparing total PIKQ-POP and PIKQ-UI scale scores and individual item scores for the patient group and the nurse group using two-tailed *t*-tests. Participants in the nurse group were more likely than those in the patient group to select the correct answer for all items for the POP scale and UI scale (Table 1). The mean total PIKQ-POP score for the nurse group was 9.28 ± 2.02 compared to 5.85 ± 2.40 for the patient group (*P* < 0.001). In agreement with the PIKQ-POP scale, the mean total PIKQ-UI score was 9.84 ± 2.16 and 6.57 ± 2.38 for the nurse group and patient group, respectively (*P* < 0.001). In addition, the median scores on the PIKQ-POP and PIKQ-UI scales across all items were significantly higher in the nurse group than in the patient group [PIKQ-POP = 10.5 (3–12) vs. 6 (0–12) and PIKQ-UI = 10 (3–12) vs. 7 (0–11), *P* < 0.001].

**Table 1:** Comparison of the mean individual item scores for the Thai PIKQ-POP and PIKQ-UI scales (*n* = 318)

Items	PIKQ-POP (mean ± SD)		PIKQ-UI (mean ± SD)	
	Patient group (n = 168)	Nurse group (n = 150)	Patient group (n = 168)	Nurse group (n = 150)
1	0.49 ± 0.50	0.82 ± 0.39	0.80 ± 0.40	0.92 ± 0.26
2	0.73 ± 0.44	0.93 ± 0.26	0.71 ± 0.46	0.87 ± 0.34
3	0.47 ± 0.50	0.61 ± 0.49	0.55 ± 0.50	0.91 ± 0.28
4	0.82 ± 0.39	0.91 ± 0.29	0.64 ± 0.48	0.80 ± 0.40
5	0.57 ± 0.49	0.79 ± 0.40	0.82 ± 0.39	0.94 ± 0.23
6	0.73 ± 0.44	0.90 ± 0.30	0.67 ± 0.47	0.93 ± 0.25
7	0.50 ± 0.50	0.77 ± 0.42	0.29 ± 0.46	0.62 ± 0.48
8	0.65 ± 0.48	0.83 ± 0.37	0.38 ± 0.49	0.80 ± 0.40
9	0.40 ± 0.49	0.77 ± 0.42	0.49 ± 0.50	0.77 ± 0.42
10	0.20 ± 0.40	0.66 ± 0.48	0.29 ± 0.45	0.74 ± 0.44
11	0.08 ± 0.27	0.67 ± 0.47	0.30 ± 0.46	0.75 ± 0.43
12	0.20 ± 0.40	0.61 ± 0.48	0.63 ± 0.49	0.75 ± 0.43

Minimum mean item score is 0, and maximum mean item score is 1

The closer the mean score is to 1, the greater percentage of patients who answer the question correctly

*PIKQ-POP:* Prolapse and Incontinence Knowledge Questionnaire-Pelvic Organ Prolapse

*PIKQ-UI:* Prolapse and Incontinence Knowledge Questionnaire-Urinary Incontinence

*SD:* standard deviation

Mann-Whitney U test

The fit indices of the PIKQ-POP and PIKQ-UI to the final one-factor model with covariance parameters are given in Table 2. All fit indices for the final models of both scales except CFI and TLI in PIKQ-UI were appropriate.

**Table 2:** Model fit statistics of the Thai PIKQ-POP and PIKQ-UI scales (*n* = 318)

Fit statistics	PIKQ-POP	PIKQ-UI
χ^2^	65.84	66.24
df	53	51
p	0.167	0.074
CFI	0.96	0.93
TLI	0.95	0.91
RMSEA (90% CI)	0.03 (0.00–0.06)	0.040 (0.000–0.07)
SRMR	0.05	0.06

*PIKQ-POP:* Prolapse and Incontinence Knowledge Questionnaire-Pelvic Organ Prolapse *PIKQ-UI:* Prolapse and Incontinence Knowledge Questionnaire-Urinary Incontinence

*Df:* degree of freedom

*CFI:* comparative fit index

*TLI:* Tucker-Lewis index

*RMSEA:* root mean square error of approximation

*CI:* confidence interval

*SRMR:* standardized root mean square residual

**Fig. 1:**
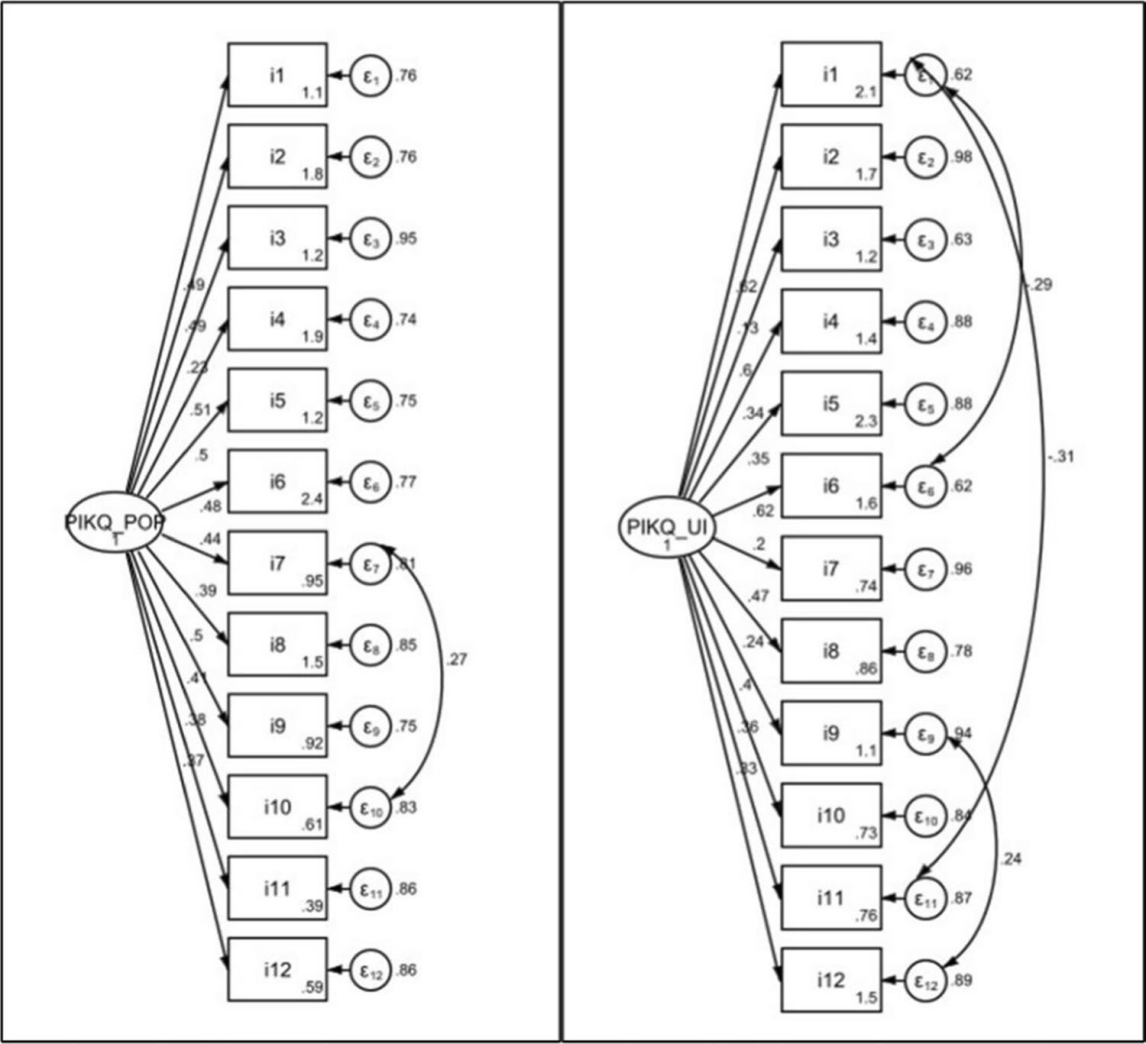
Path diagrams of final models for the Thai PIKQ-POP and PIKQ-UI scales

Table 3 shows the CFA results of the Thai PIKQ-POP and PIKQ-UI scales. While all the standardized factor loadings (SFLs) for UI items were in the acceptable range (at least 0.348), SFLs of POP items i10, i11 and i12 were < 0.30. From these results, the one-factor model was created as the best fit for the data (Fig. 1).

**Table 3:** CFA (confirmatory factor analysis) results of the Thai PIKQ-POP and PIKQ-UI scales (*n* = 318)

Items	PIKQ-POP		PIKQ-UI	
	FL ± SE	SFL ± SE	FL ± SE	SFL ± SE
1	1.000	0.551 ± 0.036	1.000	0.818 ± 0.028
2	0.823 ± 0.181	0.754 ± 0.031	0.236 ± 0.157	0.733 ± 0.032
3	0.446 ± 0.175	0.578 ± 0.036	1.250 ± 0.222	0.594 ± 0.036
4	0.825 ± 0.178	0.781 ± 0.030	0.676 ± 0.182	0.663 ± 0.035
5	0.913 ± 0.228	0.599 ± 0.036	0.529 ± 0.137	0.845 ± 0.026
6	0.657 ± 0.148	0.856 ± 0.026	1.176 ± 0.224	0.717 ± 0.033
7	0.954 ± 0.202	0.476 ± 0.037	0.399 ± 0.177	0.352 ± 0.035
8	0.682 ± 0.180	0.684 ± 0.034	0.976 ± 0.203	0.428 ± 0.036
9	0.986 ± 0.214	0.460 ± 0.036	0.500 ± 0.190	0.535 ± 0.036
10	0.807 ± 0.176	0.273 ± 0.033	0.807 ± 0.196	0.348 ± 0.035
11	0.502 ± 0.130	0.134 ± 0.025	0.730 ± 0.215	0.369 ± 0.035
12	0.620 ± 0.168	0.257 ± 0.032	0.638 ± 0.179	0.690 ± 0.034
Covariance				
i7-i10	0.050 ± 0.016		i1-i6	-0.031 ± 0.011
			i1-i11	-0.417 ± 0.013
			i9-i12	0.052 ± 0.016

*PIKQ-POP:* Prolapse and Incontinence Knowledge Questionnaire-Pelvic Organ Prolapse

*PIKQ-UI:* Prolapse and Incontinence Knowledge Questionnaire-Urinary Incontinence

*FL:* factor loading

*SFL:* standardized factor loading

*SE:* standard error

*I:* Item

#### Internal consistency (reliability)

For the internal consistency testing, the Cronbach α of each item in both scales was always above the acceptable threshold. The Cronbach’s alpha coefficient was 0.745 for PIKQ-POP and 0.754 for PIKQ-UI scales, suggesting that the items had relatively high internal consistency (Table 4). The item-total correlation values ranged from 0.204 to 0.539, showing an adequate correlation of each item with the scale overall.

**Table 4:** Internal consistency of the Thai PIKQ-POP and PIKQ-UI scales (*n* = 318)

Item number	PIKQ-POP		PIKQ-UI	
	Corrected item total correlation	Cronbach’s alpha if item deleted	Corrected item total correlation	Cronbach’s alpha if item deleted
1	0.365	0.730	0.326	0.744
2	0.440	0.723	0.252	0.752
3	0.204	0.751	0.502	0.724
4	0.307	0.736	0.357	0.741
5	0.349	0.732	0.362	0.741
6	0.336	0.733	0.497	0.726
7	0.383	0.728	0.273	0.752
8	0.343	0.732	0.461	0.728
9	0.467	0.716	0.405	0.735
10	0.420	0.723	0.539	0.717
11	0.516	0.710	0.464	0.728
12	0.430	0.722	0.243	0.755

*PIKQ-POP:* Prolapse and Incontinence Knowledge Questionnaire-Pelvic Organ Prolapse

*PIKQ-UI:* Prolapse and Incontinence Knowledge Questionnaire-Urinary Incontinence

#### Test-retest reliability (stability)

The test-retest reliability of the questionnaire was determined using the ICC score. The nurse group was invited to participate in this process. The Thai PIKQ was re-administered after an interval of 10–14 days, and 35 participants completed the questionnaire. The ICC showed a moderate-to-good test–retest reliability for both scales. Data derived from this analysis are presented in Table 5.

**Table 5:** Test-retest reliability analysis of the Thai PIKQ (*n* = 35)

	Test-retest correlation coefficient (r)	95% confidence interval	*P* value
PIKQ-POP	0.685	0.519 – 0.816	< 0.001
PIKQ-UI	0.735	0.538 – 0.872	< 0.001

*PIKQ-POP:* Prolapse and Incontinence Knowledge Questionnaire-Pelvic Organ Prolapse

*PIKQ-UI:* Prolapse and Incontinence Knowledge Questionnaire-Urinary Incontinence

## Discussion

The PIKQ is a self-administered questionnaire which has been proven to be valid and reliable to assess patient knowledge regarding POP and UI [12]. It was developed and validated originally in English language and has been widely adapted for use in different countries [25–27]. To the best of our knowledge, this is the first study with respect to validation of the PIKQ in Thai-speaking population. The findings of the present study showed that the Thai version of the PIKQ is a valid and reliable instrument, which can be used to assess knowledge of Thai-speaking women regarding POP and UI.

Questionnaire surveys are a technique for gathering statistical information aimed to measure respondents’ self-reported knowledge, attitudes, opinions or behaviors. In knowledge assessment using a questionnaire, a standard translation and cultural adaptation including an analysis of the cognitive debriefing results against the original language of the assessment tool are required. Psychometric properties of the translated version of each instrument should also be adequately assessed to confirm its validity and reliability. Since the principles of good practice for the translation and cultural adaptation process were followed methodically, it could be ensured that the Thai PIKQ questionnaire was suitable for both healthcare and non-healthcare professions [13]. Content validity assessment from the pilot testing indicated that the Thai version is equivalent to the original version of PIKQ without semantic problems. Furthermore, the missing value which diminished interpretability of the items was not found [14]. It appears that the items were clear enough to be understood by average Thai-speaking respondents aged from 20 to 79 years old.

Basically, a series of related items covering different aspects of the construct of interest should be included to confirm the construct validity of a questionnaire. The aim is to underline the extent to which the survey measures the theoretical construct it is intended to measure. To do so, the confirmatory factor analysis or other methods based on the theoretically derived hypotheses should be used to examine the construct validity of the scales [28]. In the present study, the homogeneous structures of the scales were assessed using CFA because the factor structure had been determined previously [14, 25]. All fit indices except CFI and TLI in PIKQ-UI (slightly lower) demonstrated a positive rating for construct validity. Based on these promising results, the Thai PIKQ would evaluate level of knowledge regarding pelvic organ prolapse and urinary incontinence as it is planned rather than measuring something else. Our results were comparable to the Turkish questionnaire that exhibited high criteria for validity, reliability, and sensitivity to change by demonstrating good to excellent psychometric properties [25]. In addition, similar to the findings of the original and the Turkish versions [12, 25], the Thai version of the scale showed that factor loading of item 11 was < 0.30, and a low item-factor loading for item 10 and 12 was also found in our study. Therefore, to maintain the originality of the questionnaire, no changes were applied. Moreover, construct validity of both PIKQ-POP and PIKQ-UI scales was established by showing that the nurse population, who had had a greater prior opportunity for pelvic floor dysfunction education achieved higher individual item and total scores on both scales compared with the patient or non-healthcare population.

The Thai version of the PIKQ was found to have high levels of reliability regarding internal consistency and stability. The correlation coefficient between the test and retest for the POP and UI scales exhibits the ability of this questionnaire to produce consistent responses from participants. A comparison between the findings obtained in previous studies and ours indicates the reliability testing results are similar to those from the English version of PIKQ [12] as well as the Turkish and Spanish versions [25, 26]. Accordingly, the Thai PIKQ can be used as a valid and reliable assessment tool to assess patient knowledge regarding POP and UI.

Fundamentally, the PIKQ was developed to identify populations with inadequate knowledge about POP and UI so that these populations can be properly and effectively educated and, as a consequence, can seek appropriate and timely medical care for these conditions. This instrument would not only be implemented in research projects but also in clinical practice for better quality of care. Educational strategies towards improving the knowledge should be evaluated using validated and reliable instruments. We believe that women with better knowledge regarding pelvic floor dysfunction will be more likely to seek proper care at an earlier stage. Additionally, better health-related knowledge predicts favorable health behavior.

The main strength of our study was the rigorous cross-cultural adaption and the validation process, which were fully compatible with the international guidelines [10]. The testing of the Thai version of PIKQ utilized a suitable sample size for the respondent-to-item ratios and satisfactory response rate was obtained. Nevertheless, several limitations of this study should be acknowledged. First, it was conducted in an academic tertiary-care hospital. Thus, this questionnaire should be applied or transferred in other settings or different population with some caution. Another limitation of this study is that it was not designed to evaluate the responsiveness to change. Further studies investigating the responsiveness of the PIKQ to change after educational activities are warranted.

## Conclusion

The Thai version of PIKQ is a simple instrument which conveys good validity and high reliability. It is equivalent to the original English version and would be a useful tool for assessing knowledge regarding POP and UI in clinical and research practice.

## References

[1] Wu JM, Vaughan CP, Goode PS, Redden DT, Burgio KL, Richter HE (2014). Prevalence and trends of symptomatic pelvic floor disorders in US women. Obstet Gynecol.

[2] Zeleke BM, Bell RJ, Billah B, Davis SR (2016). Symptomatic pelvic floor disorders in community-dwelling older Australian women. Maturitas.

[3] Islam RM, Oldroyd J, Rana J, Romero L, Karim MN (2019). Prevalence of symptomatic pelvic floor disorders in community-dwelling women in low and middle-income countries: a systematic review and meta-analysis. Int Urogynecol J.

[4] Fultz NH, Herzog AR (1996). Epidemiology of urinary symptoms in the geriatric population. Urol Clin North Am.

[5] Farage MA, Miller KW, Berardesca E, Maibach HI (2008). Psychosocial and societal burden of incontinence in the aged population: a review. Arch Gynecol Obstet.

[6] Ghetti C, Lee M, Oliphant S, Okun M, Lowder JL (2015). Sleep quality in women seeking care for pelvic organ prolapse. Maturitas.

[7] Geoffrion R, Robert M, Ross S, van Heerden D, Neustaedter G, Tang S (2009). Evaluating patient learning after an educational program for women with incontinence and pelvic organ prolapse. Int Urogynecol J Pelvic Floor Dysfunct.

[8] Seim A, Sivertsen B, Eriksen BC, Hunskaar S (1996). Treatment of urinary incontinence in women in general practice: observational study. BMJ.

[9] Davis K, Kumar D (2003). Pelvic floor dysfunction: a conceptual framework for collaborative patient-centred care. J Adv Nurs.

[10] Fante JF, Silva TD, Mateus-Vasconcelos ECL, Ferreira CHJ, Brito LGO (2019). Do women have adequate knowledge about pelvic floor dysfunctions? A systematic review. Rev Bras Gynecol Obstet.

[11] Branch LG, Walker LA, Wetle TT, DuBeau CE, Resnick NM (1994). Urinary incontinence knowledge among community-dwelling people 65 years of age and older. J Am Geriatr Soc.

[12] Shah AD, Massagli MP, Kohli N, Rajan SS, Braaten KP, Hoyte L (2008). A reliable, valid instrument to assess patient knowledge about urinary incontinence and pelvic organ prolapse. Int Urogynecol J Pelvic Floor Dysfunct.

[13] Wild D, Grove A, Martin M, Eremenco S, McElroy S, Verjee-Lorenz A, et al; ISPOR Task Force for Translation and Cultural Adaptation. Principles of good practice for the translation and cultural adaptation process for Patient-Reported Outcomes (PRO) Measures: report of the ISPOR Task Force for Translation and Cultural Adaptation. Value Health. 2005;8(2):94–104. 10.1111/j.1524-4733.2005.04054.x. 10.1111/j.1524-4733.2005.04054.x 15804318

[14] Terwee CB, Bot SD, de Boer MR, van der Windt DA, Knol DL, Dekker J (2007). Quality criteria were proposed for measurement properties of health status questionnaires. J Clin Epidemiol.

[15] McDonald RP, Ho MH (2002). Principles and practice in reporting structural equation analyses. Psychol Methods.

[16] Hu L, Bentler PM (1999). Cutoff criteria for fit indexes in covariance structure analysis: conventional criteria versus new alternatives. Struct Equ Model.

[17] Hu L, Bentler PM (1998). Fit indices in covariance structure modeling: Sensitivity to underparameterized model misspecification. Psychol Methods.

[18] Nunnally JC, Bernstein IH (1994). Psychometric theory.

[19] Ferketich S (1991). Focus on psychometrics. Aspects of item analysis. Res Nurs Health..

[20] Koo TK, Li MY (2016). A guideline of selecting and reporting intraclass correlation coefficients for reliability research. J Chiropr Med.

[21] Anthoine E, Moret L, Regnault A, Sébille V, Hardouin JB (2014). Sample size used to validate a scale: a review of publications on newly-developed patient reported outcomes measures. Health Qual Life Outcomes.

[22] Tinsley HE, Tinsley DJ (1987). Uses of factor analysis in counseling psychology research. J Couns Psychol.

[23] Comrey AL, Lee HB. A first course in factor analysis. Abingdon: Psychology Press; 2013. 10.4324/9781315827506.

[24] Comrey AL (1988). Factor-analytic methods of scale development in personality and clinical psychology. J Consult Clin Psychol.

[25] Toprak Celenay S, Coban O, Sahbaz Pirincci C, Korkut Z, Birben T, Alkan A (2019). Turkish translation of the Prolapse and Incontinence Knowledge Questionnaire: validity and reliability. Int Urogynecol J.

[26] Sánchez-Sánchez B, Arranz-Martín B, Navarro-Brazález B, Vergara-Pérez F, Bailón-Cerezo J, Torres-Lacomba M (2021). How do we assess patient skills in a competence-based program? Assessment of patient competences using the Spanish version of the Prolapse and Incontinence Knowledge Questionnaire and real practical cases in women with pelvic floor disorders. Int J Environ Res Public Health.

[27] Liebergall-Wischnitzer M, Cnaan T, Hochner H, Paltiel O (2015). Self-reported prevalence of and knowledge about urinary incontinence among community-dwelling Israeli women of child-bearing age. J Wound Ostomy Continence Nurs.

[28] Brown TA (2015). Confirmatory factor analysis for applied research.

